# Is olfactory reference syndrome an OCD?

**DOI:** 10.4103/0019-5545.31588

**Published:** 2006

**Authors:** Gurvinder Pal Singh

**Affiliations:** * Senior lecturer, Department of Psychiatry, Government Medical College and Hospital, Chandigarh; *mailing address:* H.No. 1202, Sector 32-B, Chandigarh 160030; e-mail: gpsluthra@rediffmail.com; gpsluthra@hotmail.com

Sir,

Although most patients with obsessive–compulsive disorder (OCD) present with classical symptoms, a range of other unusual symptoms is also seen in clinical practice. The term olfactory reference syndrome (ORS) was introduced by Pryse-Phillips and falls under the rubric of unusual obsessions.^1^ ORS can be defined as a psychiatric disorder characterized by preoccupation with body odour accompanied by significant distress and functional impairment. This syndrome comes within the spectrum of different anxiety disorders that include OCD, body dysmorphic disorder (BDD), hypochondriasis, avoidant personality disorder and taijin kyofu (a culture-bound syndrome). This condition can present as a diagnostic dilemma in clinical practice. I describe such a case below.

A 44-year-old, fair complexioned woman presented with a 17-year history of persistent preoccupation with body odour and foul smell. This smell originated from her armpits, inguinal area and the feet. She actively disguised her symptoms to prevent discovery and was ashamed of her illness. She acknowledged that these thoughts were egodystonic and distressing and that she felt like ending her life. She held herself responsible for the odour and tended to wash herself excessively by repetitive bathing and hand washing. She used to change her clothes with more than usual frequency and restricted her domestic and social outings. There were no symptoms of sadness and suicidal ideation in the absence of these repetitive thoughts. She had a family history of OCD—her father had been under treatment from a private psychiatrist. The patient had consulted many dermatologists, medical specialists, gynaecologists and had undergone multiple investigations and had received numerous treatments but did not report any notable improvement. Diagnostic criteria of different organic conditions such as severe depressive episode, OCD with psychotic features, BDD, social anxiety disorder, culture-bound syndrome and psychosis NOS (not otherwise specified) were considered. The patient's symptoms had sufficient overlap with clinical features of several psychiatric disorders. This patient did not completely fulfil the criteria for any of these disorders but fulfilled the proposed diagnostic criteria of the ORS.^2^ Her total score on the Yale-Brown Obsessive Compulsive Scale (Y-BOCS) rating was 37.^3^

The patient was treated with tablet clomipramine (75 mg daily) for 1 week and was stabilized on a daily dose of 200 mg over the next 6 weeks. Cognitive-behavioural therapy (thought stopping, Jacobson progressive muscle relaxation and distrac-tion technique) sessions were held regularly. The combined therapy showed considerable improvement in her symptoms and on her last visit the Y-BOCS total score was 10. Her preoccupation about the odour diminished in intensity and she started participating in various social activities.

The symptoms of our patient are consistent with other reported cases in the literature.^2^,^4^–^6^ ORS is not included in the ICD-10 or DSM-IV as a separate category and is perhaps most reminiscent of the BDD.^2^ In the western literature, ORS is characterized as preoccupation with the idea that the body emits a foul odour. Japanese patients with a feature similar to ORS have long been recognized as jiko-shu-kyofu, which is believed to be a culture-bound syndrome specific to Japan.^6^ Though the focus of patients with BDD is by definition on physical appearance, data exist on patients having obsessional concerns about odour.^7^ Given the considerable overlap between ORS and BDD, one can postulate that ORS is a variant of BDD and that the diagnostic criteria of BDD should be extended to include odour.

This patient presented with variable obsessive symptoms and, on detailed evaluation, other systemic and psychiatric disorders were ruled out. Increased awareness about unusual obsessions is the key for better recognition and treatment of such symptoms. The patient had a cluster of symptoms of different psychiatric disorders but did not fulfil the required diagnostic criteria for any one of them. The patient had symptoms related to body odour only. In contrast, patients with OCD generally have multiple symptoms over time. The phenomenological distinction between obsession and delusion is fairly obvious. However, in some cases the presentation is confusing. Is ORS a variant of the OCD spectrum or a separate psychotic entity? This question raises many treatment implications for such patients. More research is warranted in the near future to clarify this diagnostic dilemma. This condition is clinically relevant and can be misdiagnosed. Practitioners in areas known to attract high numbers of patients with OCD should be primed to look for the ORS. Thus, active screening should be incorporated into clinical practice. Untreated, this condition could be responsible for considerable social and emotional impairment to such patients.
